# Nano-Crystalline Li_1.2_Mn_0.6_Ni_0.2_O_2_ Prepared via Amorphous Complex Precursor and Its Electrochemical Performances as Cathode Material for Lithium-Ion Batteries

**DOI:** 10.3390/ma9080661

**Published:** 2016-08-05

**Authors:** Xiangming He, Jixian Wang, Li Wang, Jianjun Li

**Affiliations:** 1 Institute of Nuclear & New Energy Technology, Tsinghua University, Beijing 100084, China; hexm@tsinghua.edu.cn (X.H.); wangjixian520@hotmail.com (J.W.); leejj@mail.tsinghua.edu.cn (J.L.); 2 State Key Laboratory of Automotive Safety and Energy, Tsinghua University, Beijing 100084, China; 3 State Key Laboratory of New Ceramic and Fine Processing, Tsinghua University, Beijing 100084, China

**Keywords:** amorphous complex, nanoparticles, rate capability, Li-rich oxide, Lithium ion batteries

## Abstract

An amorphous complex precursor with uniform Mn/Ni cation distribution is attempted for preparing a nano-structured layered Li-rich oxide (Li_1.2_Mn_0.6_Ni_0.2_O_2_)cathode material, using diethylenetriaminepentaacetic acid (DTPA) as a chelating agent. The materials are characterized by powder X-ray diffraction (XRD), scanning electron microscopy (SEM), transmission electron microscopy (TEM), and electrochemical tests. The crystal structure of Li-rich materials is found to be closely related to synthesis temperature. As-obtained nano materials sintered at 850 °C for 10 h show an average size of 200 nm with a single crystal phase and good crystallinity. At a current density of 20 mA·g^−1^, the specific discharge capacity reaches 221 mAh·g^−1^ for the first cycle and the capacity retention is 81% over 50 cycles. Even at a current density of 1000 mA·g^−1^, the capacity is as high as 118 mAh·g^−1^. The enhanced rate capability can be ascribed to the nano-sized morphology and good crystal structure.

## 1. Introduction

Lithium-ion batteries are considered as a potential candidate for the next generation of energy storage system [1,2,3,4]. With the rapid development of the hybrid electric vehicle (HEV) and plug-in hybrid electric vehicle (PHEV), new battery chemistry with higher energy density, longer calendar life, higher reliability, and lower cost attracts increasing attention [5]. Layered LiCoO_2_ cathode material suffers from high cost, poor safety, and limited cycling life. Spinel LiMn_2_O_4_ and olivine LiFePO_4_ are relatively safe, and are proved to be applicable for electric buses, while their application in passenger cars are debatable due to their low energy densities [3,6,7]. Layered Li-rich oxides with a formula of Li[Li_(1/3-2x/3)_Mn_(2/3-x/3)_M_x_]O_2_ (M refers to Ni, Co, Mn, etc.) are regarded as promising candidates for the next generation of cathode materials due to their high capacity (200–300 mAh·g^−1^, depending on x), high operating voltage (that is higher than 3.5 V vs. Li^+^/Li), better safety, and reduced cost [8,9,10,11,12].

Despite delivering high capacity, layered Li-rich oxide materials suffer from voltage fading and poor rate capability [13,14,15], and their electrochemical performances are sensitive to the synthesis method and condition [13,16,17,18]. The layered-to-spinel transformation has been proposed to account for the capacity fading and poor rate performance [13,16,17,18,19,20]. However, the structure complexity of layered Li-rich materials has not been fully understood until now. Dahn et al. considered it as solid solution with long-range order in transition metal layer [21], while Thackeray et al. yielded a composite structure with domains only having short-range order [22]. Besides, Manthiram et al. investigated the effect of synthesis conditions on structure and electrochemical properties and reported the presence of a C2/m and R-3m two-phase mixture for samples synthesized at 1000 °C [23]. In the previous reports, many methods, including solid state method [24], sol-gel method [25], hydrothermal method [26], and combustion method [27] have been attempted to achieve Li-rich materials. To minimize layered Li-rich oxide primary particles to nano-size is generally considered to be necessary for achieving acceptable electrochemical performance [28], which means a short Li^+^ diffusion pathway and a large contact area between electrode and electrolyte [29]. It has also been accepted that different synthesis strategies show different controllability in primary particle size and morphology [24,25,30]. In addition, element segregation at particle surface was reported as being possibly responsible for performance variation of nano-size layered Li-rich oxides. Uniform distributions of the Ni element at atomic level are reported to contribute to slow voltage fading, as Zheng et al. found in Li[Li_0.2_Ni_0.2_M_0.6_]O_2_ prepared by a hydrothermal assisted sol-gel method [31]. Furthermore, Gu et al. discovered that Ni ions preferred to be segregated near the surface of Li[Li_0.2_Ni_0.2_Mn_0.6_]O_2_ particles during high temperature sintering, which may have negative effects on cycling stability and rate capability of the cathode materials [32,33,34]. They found that nanoscale segregation driven by thermodynamic force was not only in Li[Li_0.2_Ni_0.2_Mn_0.6_]O_2_ materials prepared by a co-precipitation method, but also in samples prepared using hydrothermal and sol-gel methods. Given that all of these syntheses employed annealing treatments, to further understand the thermal stability of the nano-crystal Li[Li_0.2_Ni_0.2_Mn_0.6_]O_2_ may deliver some insightful information.

The amorphous complex method is of great advantage in the synthesis of composite oxides in terms of small particle size, high crystallinity, and high phase purity at a low calcination temperature [35,36,37]. Chelating agents such as glycine have been reported to be used in the synthesis of layered LiNi_0.5_Mn_0.5_O_2_ oxide, but the decomposition of the precursor and formation of preliminary LiNi_0.5_Mn_0.5_O_2_ oxide are clearly two separate processes, which might cause nonuniform cation diffusion and cation segregation during solid state reaction processes [38]. Diethylenetriaminepentaacetic acid (DTPA) is a kind of polynuclear complex, which has been reported to be efficient in synthesizing uniform composite oxide nano-crystalline even at low temperatures [37,39,40,41]. In previous researches using DTPA as a chelating agent, decomposition of the precursor and formation of preliminary oxide particles always occur nearly simultaneously [37,40], which may avoid nonuniform solid diffusion during crystalline formation. Therefore, here we used a DTPA-based amorphous complex method to prepare nano-sized Li_1.2_Mn_0.6_Ni_0.2_O_2_ materials with uniform composition from low to high temperatures. Better understanding of the effect that temperature has on crystalline structure features, crystallization variation, and crystalline thermal stability of Li-rich materials, as well as the contribution of these characteristics to the electrochemical performances, are expected to be obtained through this study.

## 2. Experimental Section

### 2.1. Preparation of Li_1.2_Mn_0.6_Ni_0.2_O_2_ Nanoparticles

First, a certain amount of DTPA was dissolved in 150 mL deionized water under continuous heating and magnetic stirring for 1 h to form solution A. Later, LiCO_3_, MnCO_3_, and NiCO_3_·2Ni(OH)_2_·4H_2_O powder were added into solution A in stoichiometric ratio. After a 2 h reaction, the obtained solution was transferred in to a Teflon-lined stainless steel autoclave and heated at 80 °C for 12 h. A clear solution was formed. This solution was evaporated to form a precursor. The as-obtained precursor was heated at 650, 750, 850, and 950 °C for 10 h, and cooled down naturally to room temperature.

### 2.2. Materials Characterization

Thermo-gravimetric (TG) analysis and differential scanning calorimetric (DSC) analyses were performed with PerkinElmer Diamond (NETZSCHSTA 409 PC/PG, Netzsch, Selb, Germany). Crystalline structure and purity were identified by X-ray diffraction (BrukerD8 Advance X-ray diffractometer in a Bragg-Brentano configuration, Karlsruhe, Germany) with Cu Kα1 and Cu Kα2 radiation. The morphologies and element distribution of particles were characterized by scanning electron microscopy (SEM, JSM-5600LV, JEOL, Tokyo, Japan) and high-resolution transmission electron microscopy (TEM, H-800, Hitachi, Tokyo, Japan).

### 2.3. Electrochemical Measurements

For the cathode preparation, cathode film was fabricated from a mixture consisting of 80 wt % active material, 10 wt % acetylene black, and 10 wt % polytetrafluoroethylene (PTFE). Then the film was cut into rounded slices, and dried at 120 °C overnight in a vacuum oven. Celgard 2400 polypropylene film (Charlotte, NC, USA) was used as separator. Ethylene carbonate/ethyl methyl carbonate/diethyl carbonate (EC:EMC:DEC = 1:1:1 by volume) solution containing 1 mol·L^−1^ LiPF_6_ was used as electrolyte. CR2032 coin-type test cells were assembled in an argon-filled glove box using Li foil as anode. The charge–discharge tests were carried out on a Land battery test system within a voltage range of 2–4.8 V vs. Li^+^/Li at room temperature. Electrochemical impedance spectrum (EIS) measurement was carried out on a CHI 660e electrochemical working station (Shanghai Chenhua Instrument Company, Shanghai, China).

## 3. Results and Discussion

### 3.1. Structures and Morphologies Characterization

**Figure 1:**
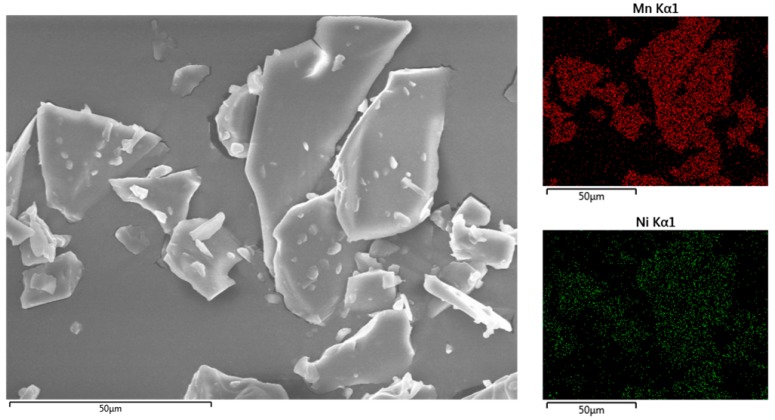
EDS mapping of the precursor.

**Figure 2:**
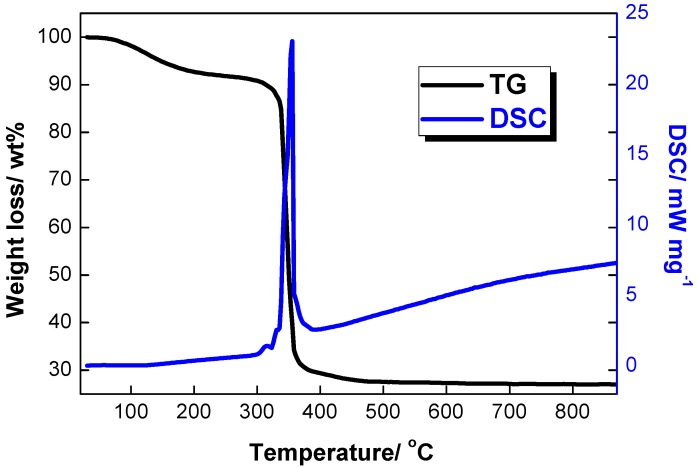
Thermo-gravimetric (TG) and DSC curves for the precursor composite at a heating rate of 4 °C·min^−1^ in flowing air.

Energy-dispersive X-ray spectroscopy (EDS) mapping was carried out to investigate the element distribution in the prepared precursor. As can be observed from Figure 1, Ni^2+^ and Mn^2+^ ions are distributed homogeneously. As shown in Figure 2, TG and DSC were used to identify the sintering temperature. The mass loss from 100 to 200 °C can be attributed to the loss of free water and crystal water from the precursor. Later, a rapid mass loss took place from 320 to 380 °C, with a sharp exothermic peak on the DSC curve. This process is corresponding to the decomposition of precursor in air and formation of preliminary particles. The last mass loss occurred from 380 to 600 °C and no obvious mass loss was observed after 600 °C. The crystallization process of layered Li-rich material may begin from about 350 °C and end at about 600 °C. Based on the above identifications, we sintered the precursor at 650, 750, 850, and 950 °C for 10 h, to optimize the synthesis temperature.

**Figure 3:**
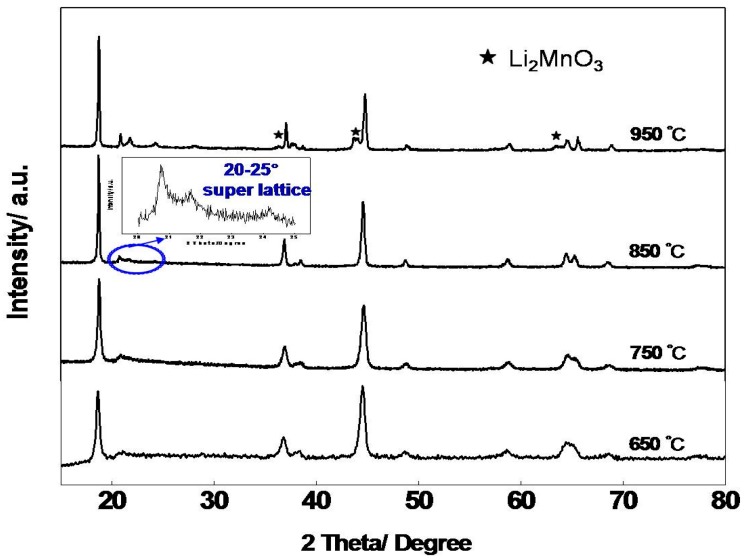
X-ray diffraction (XRD) patterns for the Li_1.2_Mn_0.6_Ni_0.2_O_2_ samples prepared at 650 °C, 750 °C, 850 °C, and 950 °C for 10 h.

Figure 3 shows the X-ray diffraction (XRD) patterns of Li_1.2_Mn_0.6_Ni_0.2_O_2_ samples prepared at 650 °C, 750 °C, 850 °C, and 950 °C for 10 h. We can see that samples sintered at 650 °C, 750 °C, and 850 °C are all indexed well in a hexagonal α-NaFeO_2_ structure, and no other impurity phase is detected [42]. With increasing sintering temperature, the intensity of the peaks increases while the full width at half-maximum(FWHM) of the reflection decreases, which means that the crystallinity of samples become higher and the crystallites grow larger. However, the sample heated at 950 °C for 10 h is accompanied by Li_2_MnO_3_ impurity. This means that at high temperatures such as 950 °C, Li-rich material is thermodynamically unstable, and preparation of layered Li-rich materials using the amorphous complex method is prone to phase segregation. Superstructure peaks around 2θ = 20°–25° results from ordering of the lithium ions and transition metal ions in the transition metal layer [43]. Furthermore, the splitting reflection peaks corresponding to (006)/(102) and (108)/(110) peak pairs are apparent for samples sintered above 750 °C, indicating layered structure is well-formed.

Since the radius of Ni^2+^ (0.69 A) is similar to that of Li^+^ (0.76 A), cation disorder may occur between Ni^2+^ and Li^+^ which may hinder Li^+^ intercalation/deintercalation during the charge/discharge process, which is harmful to material’s electrochemical performances, especially rate capability. According to previous reports, intensity ratio in XRD profile I(003)/I(104) generally reflects Li^+^/Ni^2+^ disorder, and a higher value means less cation mixing [44]. Moreover, the intensity ratio (I(006) + I(102))/I(101) can also indicate the hexagonal ordering, and a lower value means higher ordering [45]. The calculation results are shown in Table 1. We can see that when sintering temperature increased from 650 to 850 °C, the value of I(003)/I(104) ratio increased from 0.92 to 1.64; (I(006) + I(102))/I(101) is calculated as 0.38, which also represents an ordered layer structure. According to the above results, the nano-sized Li_1.2_Mn_0.6_Ni_0.2_O_2_ samples sintered at 850 °C have a well-formed hexagonal layer structure and less Li^+^/Ni^2+^ disorder, which may exhibit better electrochemical performances, especially rate capability.

**Figure 4:**
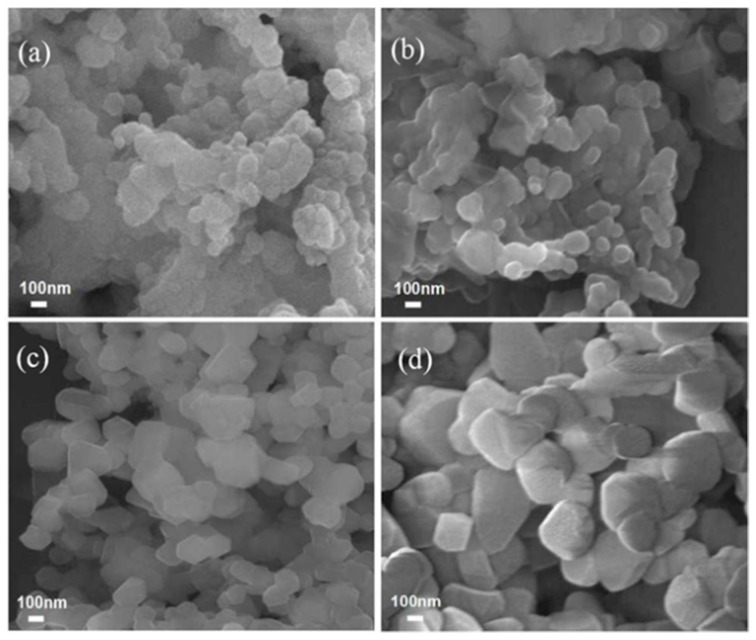
SEM images for the Li_1.2_Mn_0.6_Ni_0.2_O_2_ samples sintered at different temperature: (**a**) 650 °C; (**b**) 750 °C; (**c**) 850 °C; and (**d**) 950 °C for 10 h.

Figure 4 shows the morphologies for the samples sintered at 650–950 °C for 10 h. All the samples sintered at 650–950 °C are nanoparticles. For the samples sintered at 650 °C and 750 °C, nanoparticles with a size of about 100 nm are accompanied by formless parts. For the samples sintered at 850 °C, a morphology of well distributed nanoparticles is formed and the average particle size is about 200 nm. When the sintering temperature increases to 950 °C, the particles grow even bigger with some agglomeration to form a network, which agrees well with analysis of XRD patterns.

**Figure 5:**
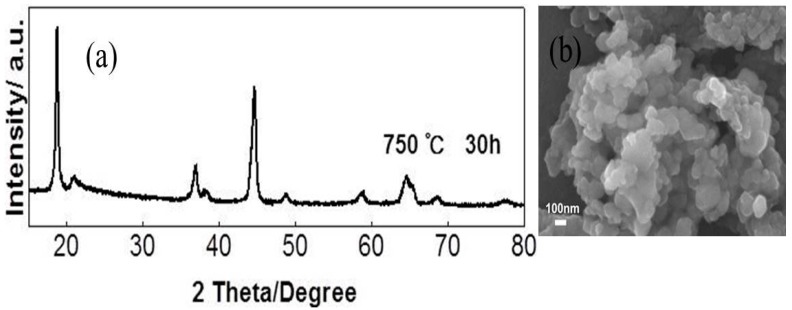
(**a**) XRD patterns; (**b**) SEM images for the Li_1.2_Mn_0.6_Ni_0.2_O_2_ sample prepared at 750 °C for 30 h.

Layered Li-rich materials are always synthesized under high temperatures (above 850 °C) for co-precipitation [42] and sol-gel preparation [25], because a lithium diffusion process is essential. However, it was reported that Ni ions prefer to be segregated near the particle surface during high temperature sintering in these synthesis processes, which might have negative effect on cycling stability and rate capability [33]. The amorphous complex method has been reported to be efficient in preparing nano-sized oxides at lower temperatures [37]. As shown in Figure 3, it can be clearly seen that layered structure material was obtained under 750 °C for 10 h, although its layered structure was not well-formed. We prolonged sintering time to 30 h under 750 °C, intending to investigate the practicality to synthesis layered Li-rich materials at lower temperatures. As shown in Figure 5a, after calcination at 750 °C for 30 h, the intensity of XRD peaks and typical splitting reflection peaks change little. Moreover, the calculation results of I(003)/I(104)and (I(006) + I(102))/I(101) are shown in Table 2, which also show little improvement. Figure 5b shows the morphology for the samples sintered at 750 °C for 30 h, which is still nanoparticles accompanied by formless parts, compared with the sample sintered at 750 °C for 10 h. Hence, it is clear that the calcination temperature of 750 °C is not sufficient for good hexagonal structure and nano-sized particle growth in the amorphous complex process.

**Figure 6:**
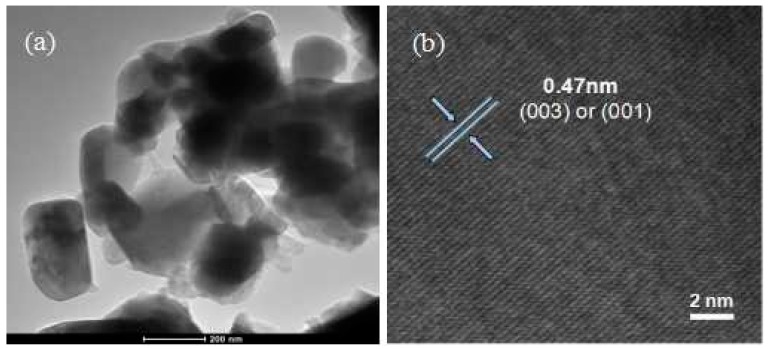
TEM (**a**) and high-resolution TEM (HRTEM); (**b**) images for the Li_1.2_Mn_0.6_Ni_0.2_O_2_ samples sintered at 850 °C for 10 h.

Microstructure of the sample sintered at 850 °C was further investigated by EDS mapping, TEM and high-resolution TEM (HRTEM). As can be clearly seen from EDS mapping spectra (Supplementary Figure S1), Mn/Ni cations are uniformly distributed in the final product. The TEM image (Figure 6a) clearly shows that sample sintered at 850 °C consists of well distributed nanoparticles with an average size of 200 nm. The fringes in HRTEM image (Figure 6b) are identified as being 0.47 nm, which agrees well with the {003} and {001} lattice spacing of rhombohedral Li*M*O_2_ (*M* = Mn, Ni, etc.). This means that the Li_1.2_Mn_0.6_Ni_0.2_O_2_ samples sintered at 850 °C for 10 h are well-crystallized and well mono-dispersed single phase nanoparticles.

### 3.2. Electrochemical Performances of Li_1.2_Mn_0.6_Ni_0.2_O_2_ Material

**Figure 7:**
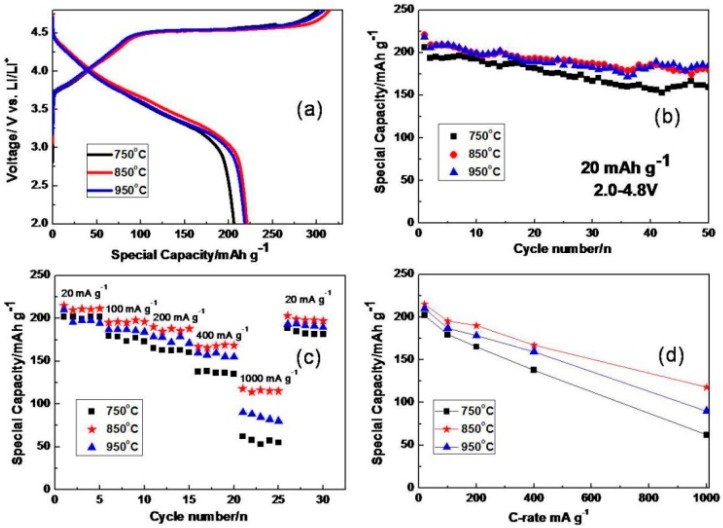
(**a**) Initial charge–discharge profiles at a current density of 20 mA·g^−1^; (**b**) cycling performance at a current density of 20 mA·g^−1^; (**c**) rate capability; and (**d**) comparison of discharge capacity at different rate.

Electrochemical performances of the samples prepared at different temperature are depicted in Figure 7. Figure 7a shows the charge/discharge profiles of the first cycle between 2.0 and 4.8 V vs. Li^+^/Li at a current density of 20 mA·g^−1^. The first region below 4.5 V is ascribed to the oxidation of Ni^2+^ to Ni^4+^, while the subsequent flat region is assigned to the removal of Li_2_O from such solid solution materials [13,42].

The initial charge and discharge capacities for the sample prepared at 750 °C are measured as 306 mAh·g^−1^ and 206 mAh·g^−1^, respectively. After 50 cycles, the discharge capacity retains at 159 mAh·g^−1^ with the lowest capacity retention of 77.4%, which means its layered structure is not so stable. For the sample sintered at 850 °C, it delivers the highest initial discharge capacity of 221 mAh·g^−1^, which decreases to 180 mAh·g^−1^ after 50 cycles with a retention of 81%. The initial discharge capacity of material synthesized at 950 °C is 218 mAh·g^−1^, which is slightly lower than that of sample prepared at 850 °C. However, it exhibits a higher capacity retention of 84% after 50 cycles. The main reason for capacity fading might also be the layer to spinel phase transformation, which we have mentioned before [19,20]. We have found that there is Li_2_MnO_3_ phase separation for material synthesized at 950 °C, and nano-sized Li_2_MnO_3_ with high crystallinity is reported to be slowly activated and exhibit somewhat stable discharge capacities at low current density [46,47], which maybe the main reason for its slightly lower initial discharge capacity.

Furthermore, to investigate the effect on kinetic behavior by reducing particle size to nanoscale, we investigated the rate capability of as-prepared samples at different current densities from 20 to 1000 mA·g^−1^. As compared in Figure 7c, the sample obtained at 850 °C shows the best rate performance. When the current density is increased to 200 mA·g^−1^, the capacity is as high as 190 mAh·g^−1^. Even when the discharge current density is increased up to 1000 mA·g^−1^, the capacity still can retain up to 118 mAh·g^−1^. Compared with some reported Li_1.2_Mn_0.6_Ni_0.2_O_2_ materials without doping [48,49], the uniform nano-sized Li_1.2_Mn_0.6_Ni_0.2_O_2_ material prepared by the amorphous complex method shows improved rate capability, which shows the benefits of reducing particle size for improving high rate performances of Li-rich materials. 

It is apparent from Figure 7d that sintering temperature influences the rate capability significantly. Considering the analysis results of XRD and SEM, the relatively low temperature of 750 °C will bring about worse crystallinity and poor structure, which will have a negative impact on Li^+^ insertion/extraction. While a high temperature of 950 °C may result in large particle size and Li_2_MnO_3_ phase segregation, the sample obtained at 850 °C shows the best crystal structure features and uniform morphology, which leads to enhanced rate capability. To better understand electrochemical properties of the materials obtained at different temperatures, EIS measurements of these three samples were conducted. 

**Figure 8:**
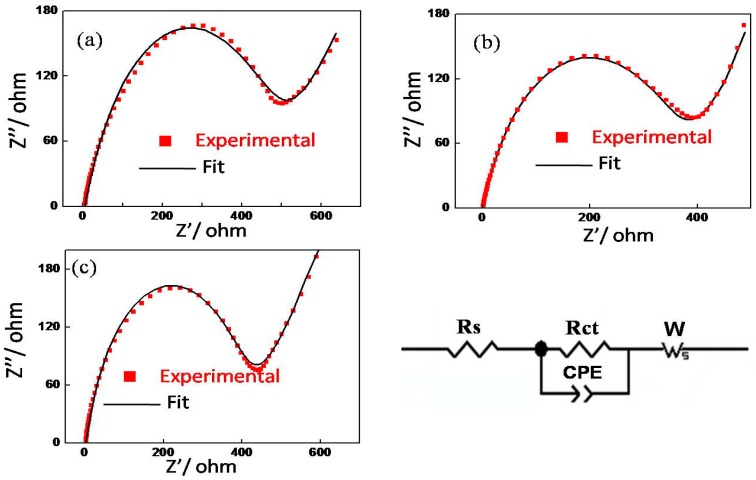
Nyquist plots of fresh cells of material sintered at (**a**) 750 °C; (**b**) 850 °C; and (**c**) 950 °C for 10 h.

Figure 8 shows Nyquist plots of fresh cells at open circuit voltages (OCV) at room temperature, right after the half-cell was assembled. All the Nyquist plots are composed of one semicircle and an inclined line. The semicircle at high frequency might be due to the R_s_ (solid electrolyte interface resistance) and R_ct_ (charge transfer resistance), and the inclined line at low frequency is attributed to Warburg impedance that is associated with Li^+^ diffusion through the cathode. R_s_ and R_ct_ can sometimes overlap, especially when the cell was not cycled [50]. For these fresh cells without cycling, the solid electrolyte interface (SEI) formation is very weak and the semicircle is mainly determined by R_ct_. As seen in Figure 8, the R_ct_ of material sintered at 850 °C (355 Ω) is smaller than that obtained from samples sintered at 750 °C (468 Ω) and 950 °C (390 Ω), which is indicative of higher electrical conductivity and faster electrochemical reactions [51]. We also evaluated the Li^+^ diffusion coefficiency at fully charged from EIS spectra using a reported method [52,53]. The results reveal that material sintered at 850 °C has a higher Li^+^ diffusion coefficiency (7.64 × 10^−13^ cm^2^·s^−1^) than samples sintered at 750 °C (3.24 × 10^−14^ cm^2^·s^−1^) and 950 °C (1.06 × 10^−13^ cm^2^·s^−1^), which may be attributed to the uniform nano-sized distribution and lower Li^+^/Ni^2+^ disorder of material sintered at 850 °C. As for material sintered at 950 °C, although its R_ct_ is only slightly greater than that of the material sintered at 850 °C, it is well known that the Li^+^ diffusion in Li_2_MnO_3_ phase is slow, and the SEM image shows that the particle size is growing even bigger, which is predicted to have a negative impact on rate capability. Overall, the EIS testing results show that sample sintered an appropriate temperature of 850 °C shows the highest electrical conductivity and rapid Li^+^ diffusion. The rapid Li^+^ diffusion might be responsible for its enhanced rate capability.

## 4. Conclusions

Well-dispersed Li_1.2_Ni_0.2_Mn_0.6_O_2_ nanoparticles with an average size of 200 nm have been prepared by a facile amorphous complex method, and the influence of sintering temperature on crystallization using an amorphous complex precursor has been studied. The relatively low temperature of 750 °C is not sufficient for obtaining well-formed hexagonal ordering and nanoparticles’ growth, while phase segregation may occur under high temperature as 950 °C, which indicates the thermodynamic instability of Li-rich materials at high temperature. The sample obtained at a sintering temperature of 850 °C shows uniform Mn/Ni cation distribution, good cycle ability, and rate capability, which maybe due to its good crystal structure and well-distributed nano morphology. This nano-sized material can deliver a capacity of 221 mAh·g^−1^ at a current density of 20 mA·g^−1^ in the potential window of 2.0–4.8 V vs. Li^+^/Li, and remains about 81% after 50 cycles. The discharge capacity can be retained as high as 118 mAh·g^−1^ even at a current density of 1000 mA·g^−1^. This study demonstrates a simple and efficient way to prepare nano-sized layered Li-rich cathode materials and shows the relationship between crystal structure and synthesis temperature. Although the as-prepared nano-sized sample sintered at 850 °C exhibits good crystal structure, uniform Mn/Ni cation distribution and improved kinetic behaviors, capacity fading still exists. Future work will be conducted to further understand the capacity fading mechanism, and use cation doping to improve its cycling stability and rate capability for further application in lithium-ion batteries.

## Figures and Tables

**Table 1:** The values of I(003)/I(104) and (I(006) + I(102))/I(101) calculated from XRD patterns.

Sample	I(003)/I(104)	(I(006) + I(102))/I(101)
650 °C	0.92	0.60
750 °C	1.33	0.50
850 °C	1.64	0.41
950 °C	1.78	0.36

**Table 2:** I(003)/I(104) and (I(006) + I(102))/I(101) for sample sintered at 750 °C for 30 h.

Sample	I(003)/I(104)	(I(006) + I(102))/I(101)
750 °C for 30 h	1.34	0.52

## Supplementary Materials

The following are available online at www.mdpi.com/1996-1944/9/8/661/s1.
