# Supplementary material for: Nano-Crystalline Li1.2Mn0.6Ni0.2O2 Prepared via Amorphous Complex Precursor and Its Electrochemical Performances as Cathode Material for Lithium-Ion Batteries
Source: Materials (Basel). 2016 Aug 5;9(8):661. doi: 10.3390/ma9080661 (PMC5509272; doi:10.3390/ma9080661)
Supplement: Supplementary file 1 [file materials-09-00661-s001.pdf]

Supplementary Materials: Nano-Crystalline $Li_{1.2}Mn_{0.6}Ni_{0.2}O_2$ Prepared via Amorphous Complex Precursor and Its Electrochemical Performances as Cathode Material for Lithium-Ion Batteries

Xiangming He, Jixian Wang, Li Wang and Jianjun Li

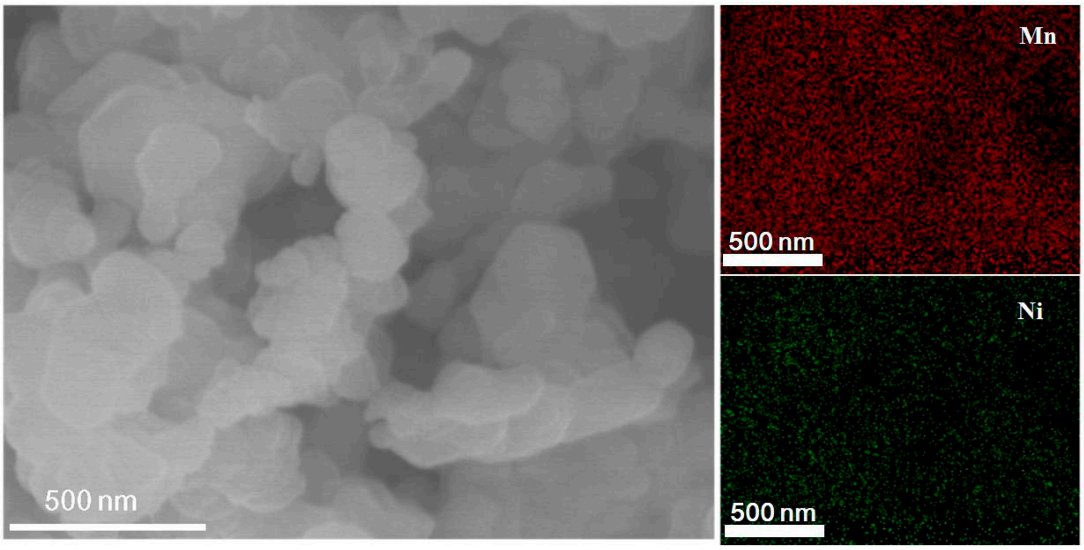

Figure S1. SEM photograph and EDS mapping spectra of $Li_{1.2}Mn_{0.6}Ni_{0.2}O_2$ particles sintered at 850 °C for 10 h.

Figure S1 shows the energy-dispersive X-ray spectroscopy (EDS) mapping analysis of the $Li_{1.2}Mn_{0.6}Ni_{0.2}O_2$ sample sintered at 850 °C for 10 h. EDS mapping verifies the uniform Mn/Ni cation distribution in the final product. The spectra illustrate that Mn/Ni cations are uniformly distributed in the final product.
